# Pharmacokinetic interaction study between ligustrazine and valsartan in rats and its potential mechanism

**DOI:** 10.1080/13880209.2020.1859554

**Published:** 2020-12-23

**Authors:** Yang Liu, Jiaqi Zhang, Di Wu, Liran Cui

**Affiliations:** a Department of Pharmacy, The First Affiliated Hospital of Qiqihar Medical University, Fularji District, Qiqihar, China; b Central Laboratory, The First Affiliated Hospital of Qiqihar Medical University, Fularji District, Qiqihar, China

**Keywords:** Metabolic stability, CYP3A4, metabolism, drug–drug interaction

## Abstract

**Context:**

Ligustrazine and valsartan are commonly used drugs in the treatment of cardiac and cardiovascular disease.

**Objective:**

The interaction between ligustrazine and valsartan was studied to investigate the effect of ligustrazine on the pharmacokinetics of valsartan.

**Materials and methods:**

The pharmacokinetics of valsartan (10 mg/kg) was investigated in Sprague–Dawley rats divided into three groups (with the pretreatment of 4 or 10 mg/kg/day ligustrazine for 10 days and without the pretreatment of ligustrazine as control) of six rats each. The *in vitro* experiments in rat liver microsomes were performed to explore the effect of ligustrazine on the metabolic stability of valsartan.

**Results:**

Ligustrazine changed the pharmacokinetic profile of valsartan. In the presence of 4 mg/kg ligustrazine, the AUC_(0–_*_t_*_)_ (385.37 ± 93.05 versus 851.64 ± 104.26 μg/L*h), *t_1/2_* (5.46 ± 0.93 versus 6.34 ± 1.25 h), and *C*_max_ (62.64 ± 9.09 versus 83.87 ± 6.15 μg/L) of valsartan was significantly decreased, and the clearance rate was increased from 10.92 ± 1.521 to 25.76 ± 6.24 L/h/kg and similar changes were observed in the group with 10 mg/kg ligustrazine (*p* < 0.05). The metabolic stability of valsartan was also decreased by ligustrazine as the half-life of valsartan in rat liver microsomes decreased from 37.12 ± 4.06 to 33.48 ± 3.56 min and the intrinsic clearance rate increased from 37.34 ± 3.84 to 41.40 ± 4.32 μL/min/mg protein (*p* < 0.05).

**Discussion and conclusions:**

Ligustrazine promoted the metabolism of valsartan via activating CYP3A4. The co-administration of ligustrazine and valsartan should be taken into account.

## Introduction

Ligustrazine is a bioactive ingredient extracted from *Rhizoma Ligustici wallichii* [*Ligustici wallichii* (Apiaceae)]. Ligustrazine has been widely used in the clinic for the treatment of cardiovascular disease because of its function in activating blood circulation (Wu et al. 2012; Qian et al. 2014). Moreover, ligustrazine has been demonstrated to possess antioxidant properties in animal models of ischemic-reperfusion, atherosclerosis, and cerebral vasospasm (Jiang et al. 2011; Lv et al. 2012). Commonly, patients with cardiovascular or cerebral diseases may have received ligustrazine treatment before using other drugs, or it may be used with other drugs together for the treatment of complex chronic disorders (Hockenberry 1991; Phougat et al. 2014).

Valsartan is a commonly used drug in cardiac disease with relatively low absolute bioavailability (Jung et al. 2015). Valsartan is usually applied in the treatment of hypertension due to its properties of lowing blood pressure, and it also co-administrated with other drugs to make the treatment more efficient in the clinic (Cheng et al. 2001; Liu et al. 2019a). Previously, the co-administration of valsartan and quercetin could exert greater cardiac protection and this kind of combination affected the pharmacokinetics of valsartan (Challa et al. 2013). The combination of valsartan and amlodipine has been demonstrated to control blood pressure efficiently and the pharmacokinetic and transport of valsartan were influenced by the administration of amlodipine (Cai et al. 2011). The combination of ligustrazine and valsartan has been reported to have a protective effect on hippocampal neuronal loss with vascular dementia (Qin et al. 2011). However, the drug–drug interaction between ligustrazine and valsartan was still unclear.

This study focussed on the interaction between ligustrazine and valsartan to evaluate the effect of ligustrazine on the pharmacokinetics of valsartan, which can provide more information for the clinical co-administration of ligustrazine and valsartan.

## Materials and methods

### Drugs and chemicals

Standards of valsartan (purity >98%), and ligustrazine (purity >98%) were purchased from the National Institute for the Control of Pharmaceutical and Biological Products (Beijing, China). β-NADPH was obtained from Sigma Chemical Co. (St. Louis, MO). Pooled RLM were purchased from BD Biosciences Discovery Labware (Woburn, MA). Acetonitrile and methanol were purchased from Fisher Scientific (Fair Lawn, NJ). Formic acid was purchased from Anaqua Chemicals Supply Inc. Limited (Houston, TX). Ultrapure water was prepared with a Milli-Q water purification system (Millipore, Billerica, MA). All other chemicals were of analytical grade or better.

### Animals

Experiments were performed with male Sprague–Dawley rats weighing 230–250 g supplied by Sino-British Sippr/BK Lab Animal Ltd (Shanghai, China). The rats were housed in an air-conditioned animal quarter at 22 ± 2 °C and 50 ± 10% relative humidity. The animals were acclimatized to the facilities for 5 days, during this period, animals had free access to food and water. Before each experiment, the rats fasted with free access to water for 12 h. The experiment was approved by the Animal Care and Use Committee of The First Affiliated Hospital of Qiqihar Medical University.

### Preparation of plasma samples

Plasma sample (100 μL) was mixed with 20 μL methanol and 180 μL internal standard methanol solution and vortexed for 60 s. After centrifugation at 12,000 rpm for 10 min, the supernatant was removed into an injection vial and 5 μL aliquot was injected into LC/MS-MS system for analysis.

### LC-MS/MS condition

Chromatographic analysis was performed by using an Agilent 1290 series liquid chromatography system (Agilent Technologies, Palo Alto, CA) as previously reported (Deng et al. 2017; Liu et al. 2019b). The sample was separated on Waters Xbridge C18 column (100 × 3.0 mm, i.d.; 3.0 μm, Waters Corporation, Milford, MA) and eluted with an isocratic mobile phase: solvent A (Water containing 0.1% formic acid) – solvent B (acetonitrile) (65:35, v/v). The column temperature was set at 25 °C, the flowing rate at 0.4 mL/min, and the injection volume at 5 μL. Mass spectrometric detection was carried out on an Agilent 6460 triple-quadruple mass spectrometer (Agilent Technologies, Palo Alto, CA) with Turbo Ion spray, which is connected to the liquid chromatography system. The mass scan mode was MRM positive. The precursor ion and product ion were *m*/*z* 434.2→179.0 for valsartan and *m*/*z* 357.9→321.8 for methyclothiazide as IS according to the previous study (Wei et al. 2019). The collision energy for valsartan and methyclothiazide was 30 and 20 eV, respectively. The MS/MS conditions were optimized as follows: fragmentor, 110 V; capillary voltage, 3.5 kV; nozzle voltage, 500 V; nebulizer gas pressure (N_2_), 40 psig; drying gas flow (N_2_), 10 L/min; gas temperature, 350 °C; sheath gas temperature, 400 °C; sheath gas flow, 11 L/min. Agilent MassHunter B.07 software was used for the control of the equipment and data acquisition. Agilent Quantitative analysis software was used for data analysis.

### Pharmacokinetic study

The rats were randomly divided into three groups of six animals each, including 10 mg/kg valsartan only group (A), 10 mg/kg valsartan + 4 mg/kg ligustrazine group (B), and 10 mg/kg valsartan + 10 mg/kg ligustrazine (C). The administration of ligustrazine was prior to valsartan for 10 days. Blood samples (0.25 mL) were collected into a heparinized tube via the oculi choroidal vein before drug administration and at 0.083, 0.25, 0.5, 1, 2, 3, 4, 6, 8, 12, and 24 h after drug administration. After centrifuging at 3500 rpm for 10 min, the supernatant was obtained and frozen at −80 °C until analysis.

### The metabolic stability of valsartan in rat liver microsomes

According to previous studies, the reaction mixture was pre-incubated at 37 °C for 5 min, after that 1 μM valsartan was added (Qi et al. 2014; Li et al. 2016). For the effect of ligustrazine on the metabolic stability of valsartan, ligustrazine was added into rat liver microsomes and pre-incubated for 30 min at 37 °C, and then valsartan was added. After incubating for 0, 1, 3, 5, 15, 30, and 60 min, 30 μL samples were collected from volumes and the reaction was terminated by ice-cold acetonitrile. Then samples were prepared according to the above methods and analyzed by LC-MS/MS.

The half-life (*t*_1/2_) *in vitro* was obtained using equation: *t*_1/2_ *= 0.693/k*. V (μL/mg) = volume of incubation (μL)/protein in the incubation (mg); Intrinsic clearance (Clint) (μL/min/mg protein) = V × 0.693/*t*_1/2_.

### Statistical analysis

All data were represented as mean ± SD obtained from triplicate experiments and analyzed with one-way analysis of variance (ANOVA) with SPSS 20.0 (SPSS, Inc., Chicago, IL). The pharmacokinetic parameters of valsartan were obtained with DAS 3.0 pharmacokinetic software (Chinese Pharmacological Association, Anhui, China). Differences were considered to be statistically significant when *p* < 0.05.

## Results

### Effect of ligustrazine on the pharmacokinetics of valsartan

**Figure 1.**
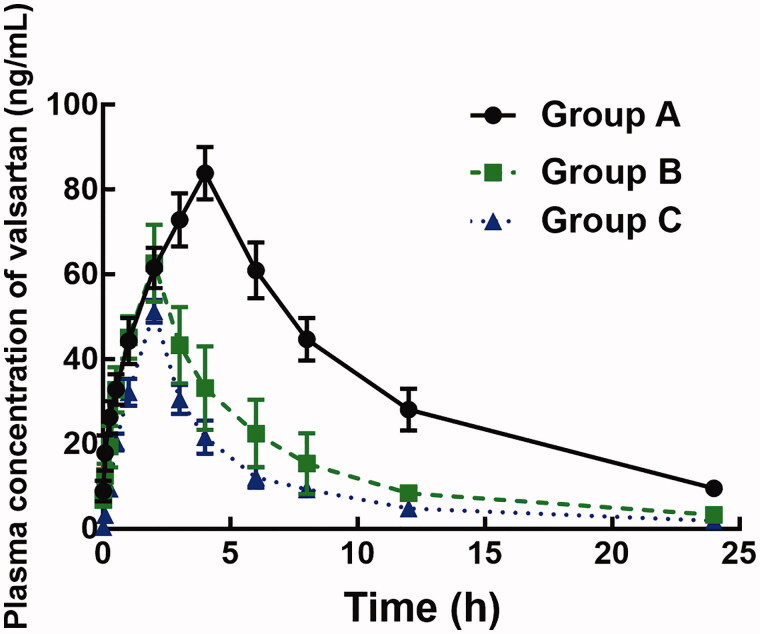
Plasma concentration–time curves of valsartan in the presence or the absence of 4 or 10 mg/kg ligustrazine.

The plasma concentration of valsartan was analyzed by LC-MS/MS (Supplementary Figure 1) and the plasma concentration–time curves of valsartan in rats with or without treatment of ligustrazine are shown in Figure 1. The pharmacokinetic parameters, including area under curves (*AUC*_(0–_*_t_*_)_), half-life (*t*_1/2_), time to reach maximum concentration (*T*_max_), maximum concentration (*C*_max_), and clearance (*Clz/F*), are summarized in Table 1.

**Table 1.** Pharmacokinetic parameters of valsartan (10 mg/kg) with or without ligustrazine (4 or 10 mg/kg).

	Valsartan	Valsartan + ligustrazine (4 mg/kg)	Valsartan + ligustrazine (10 mg/kg)
AUC_(0-t)_ (μg/L*h)	851.64 ± 104.26	385.37 ± 93.05*	249.85 ± 41.27*
t_1/2_ (h)	6.34 ± 1.25	5.46 ± 0.93*	5.15 ± 0.88*
T_max_ (h)	3.88 ± 0.13	1.79 ± 0.22*	1.83 ± 0.19*
C_max_ (μg/L)	83.87 ± 6.15	62.64 ± 9.09*	51.28 ± 2.67*
Clz/F (L/h/kg)	10.92 ± 1.52	25.76 ± 6.24*	39.44 ± 6.53*

* *p* < 0.05.

The administration of ligustrazine significantly changed the pharmacokinetic profile of valsartan. In the presence of 4 mg/kg ligustrazine, the *AUC*_(0–_*_t_*_)_ of valsartan decreased from 851.64 ± 104.26 to 385.37 ± 93.05 μg/L/h and the *C*_max_ reduced from 83.87 ± 6.15 to 62.64 ± 9.09 μg/L (*p* < 0.05). Similarly, the oral administration of 10 mg/kg ligustrazine also dramatically decreased the AUC_(0–_*_t_*) (249.85 ± 41.27 versus 851.64 ± 104.26 μg/L/h) and *C*_max_ (51.28 ± 2.67 versus 83.87 ± 6.15 μg/L). The *t*_1/2_ of valsartan reduced from 6.34 ± 1.25 to 5.46 ± 0.93 h after co-administrated of 4 mg/kg ligustrazine and to 5.15 ± 0.88 h in the presence of 10 mg/kg ligustrazine, and the difference was significant (*p* < 0.05). Additionally, the clearance rate of valsartan was significantly enhanced by both dose of ligustrazine (from 10.92 ± 1.521 to 25.76 ± 6.24 L/h/kg for 4 mg/kg ligustrazine, and to 39.44 ± 6.53 L/h/kg for 10 mg/kg ligustrazine, *p* < 0.05, Table 1).

### Effect of ligustrazine on the metabolic stability of valsartan

In rat liver microsomes, the obtained half-life of valsartan was 37.12 ± 4.06 min, which was significantly shortened to 33.48 ± 3.56 min after the administration of ligustrazine (*p* < 0.05). Moreover, the intrinsic clearance rate of valsartan significantly increased from 37.34 ± 3.84 to 41.40 ± 4.32 μL/min/mg protein by ligustrazine indicating the significant decrease in the metabolic stability of valsartan after the co-administration with ligustrazine (*p* < 0.05).

## Discussion

Both ligustrazine and valsartan are widely used in the therapy of cardiac and cardiovascular disease. A combination of ligustrazine and valsartan can exert protective effects on the hippocampal neuronal loss with vascular dementia (Qin et al. 2011). Previous studies reported the co-administration of different drugs could induce adverse interaction results in the induction or inhibition of drug pharmacokinetics. For example, puerarin could inhibit the metabolism of triptolide and increase the plasma concentration of triptolide, which would increase the toxicity of triptolide (Wang et al. 2019). Therefore, it is necessary to investigate the interaction between different drugs.

The co-administration of ligustrazine and valsartan investigated in this study indicated the promoted effect of ligustrazine on the metabolism of valsartan. Co-administration of 4 or 10 mg/kg ligustrazine and 10 mg/kg valsartan significantly reduced the AUC_(0–_*_t_*_)_, *t*_1/2_, and *C*_max_ of valsartan in rat and increased the clearance rate of valsartan. For *in vitro* experiments, the metabolic stability of valsartan in rat liver microsomes was decreased by ligustrazine with the increased intrinsic clearance rate and decreased half-life. According to previous studies, the metabolism of valsartan is mainly mediated by CYP3A4, which is an important enzyme that participates in the phase-I metabolism of various drugs in the liver (Nakashima et al. 2005; Manikandan and Nagini 2018). Ligustrazine was also demonstrated to be an inducer of CYP3A4 (Zhang et al. 2014). The activity of CYP3A4 plays a vital role in the drug–drug interaction between valsartan and many other co-administrated drugs, such as quercetin (Challa et al. 2013). It also induced the drug-drug interaction between other drugs. For example, the co-administration of glycyrrhizin and nobiletin led to the enhanced metabolism of nobiletin via inducing the activity of CYP3A4 (Wang et al. 2020). Therefore, it is speculated that the effect of ligustrazine on the pharmacokinetics of valsartan was a result of the enhanced activity of CYP3A4. However, the concentration of drugs during drug–drug interaction is an important factor. The induced effect of ligustrazine on the activity of CYP3A4 was reported at the ligustrazine concentration of 20 μM (Zhang et al. 2014), which is much higher than the dosage used in this study. Therefore, it is necessary to validate the effect of ligustrazine on the activity of CYPs at different concentrations.

This study provides direct *in vivo* and *in vitro* evidence to prove the promoted effect of ligustrazine on the pharmacokinetics of valsartan. The interaction between these two drugs in humans needs to be explore, which should be studied with more experiments closer to the metabolism in humans. Moreover, the effect of ligustrazine on the pharmacokinetics of valsartan resulted from the induction of CYP3A4. Hence, valsartan should be carefully used with drugs that affect the activity of CYP3A4.

## Conclusions

The co-administration of valsartan and ligustrazine induced drug–drug interaction, which decreased the system exposure of valsartan by activating CYP3A4. Therefore, the dose of ligustrazine and valsartan should be carefully considered in the clinic, when they are co-administrated.

## Supplementary Material

Supplemental Material
