# Supplementary figures and images for: Pharmacokinetic interaction study between ligustrazine and valsartan in rats and its potential mechanism
Source: Pharm Biol. 2020 Dec 23;58(1):1299–302. doi: 10.1080/13880209.2020.1859554 (PMC7759250; doi:10.1080/13880209.2020.1859554)

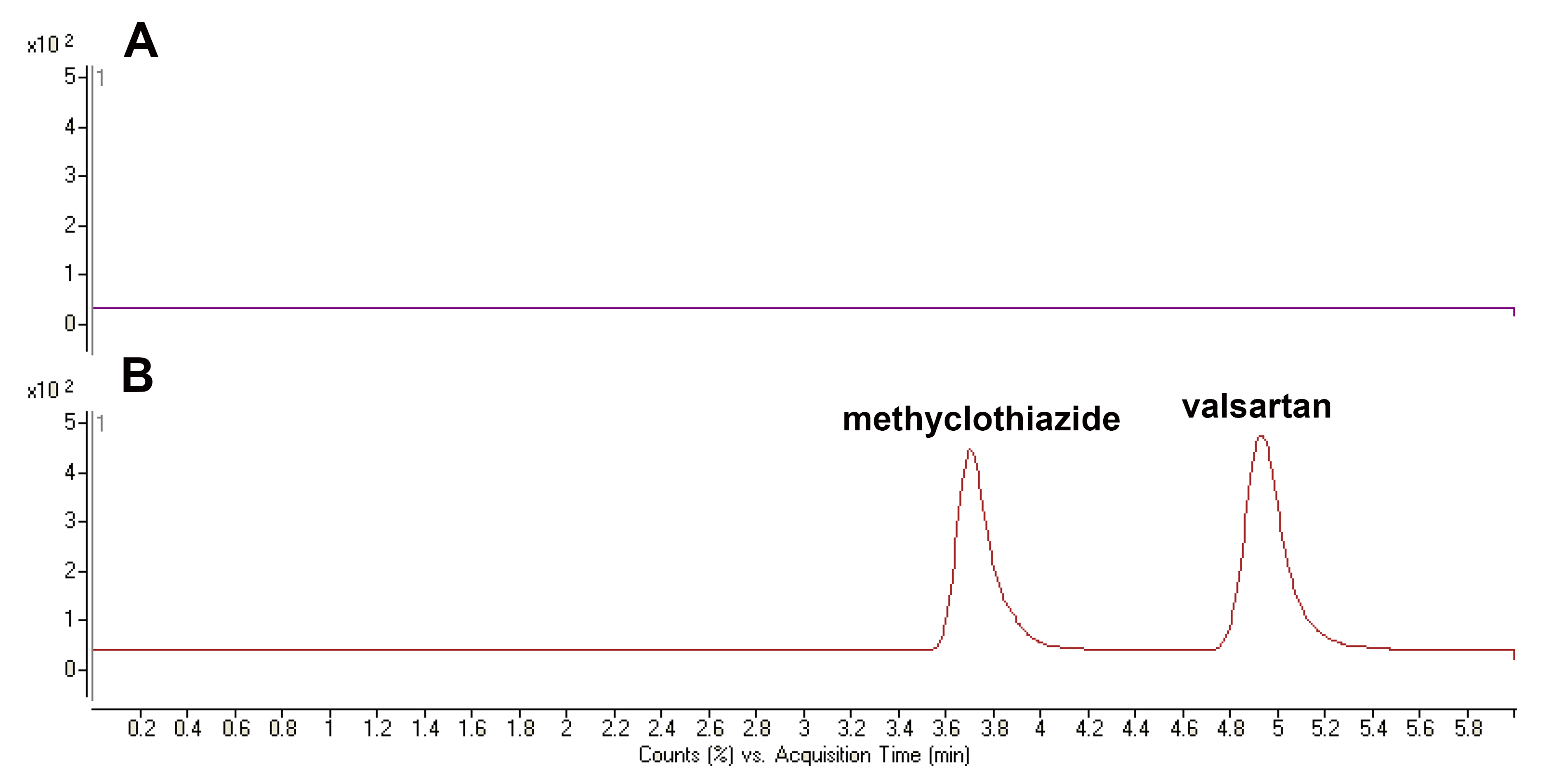

Supplement: Supplemental Material [file IPHB_A_1859554_SM4930.tif]
